# How Team Structure Can Enhance Performance: Team Longevity’s Moderating Effect and Team Coordination’s Mediating Effect

**DOI:** 10.3389/fpsyg.2020.01873

**Published:** 2020-07-31

**Authors:** Hao Ji, Jin Yan

**Affiliations:** ^1^Business School, Ningbo University, Ningbo, China; ^2^School of Management, Zhejiang University, Hangzhou, China

**Keywords:** team structure, team coordination, team longevity, team performance, team learning

## Abstract

Teams are more or less structured in function. Whether team structure is beneficial or harmful for the teams entail debates in current literature. Past studies mainly investigate the effects of team structure through learning or creativity. In this study, we tend to examine the effect of team structure on team performance through team coordination. We conducted two independent field studies with samples of 56 and 67 work teams to test our hypotheses. In both two substudies, we found team structure positively affect team performance by improving team coordination. Moreover, we found team longevity was able to moderate the relationship between team structure and team performance through team coordination, such that the positive relationship between team structure and team coordination were more significant when team longevity was high rather than low.

## Introduction

Teams have been considered as fundamental units in today’s organizations (Mathieu et al., 2014). As a substitution for highly structured departments, teams have been traditionally considered to function without a structure (e.g., Mintzberg, 1979). However, recent studies suggest that teams usually employ structural elements to guide or coordinate their work. For instance, they are likely to elect a leader to monitor individual team member work, divide collective work among team members, and set rules or procedures for teamwork, including deadlines for tasks (e.g., Langfred, 2007; Bunderson and Boumgarden, 2010; Conaldi and Lomi, 2013; Meyer et al., 2017). These structural elements have been defined as team structure, which refers to the extent to which specialization, hierarchy and routines and/or rules are clearly defined within the team (Bunderson and Boumgarden, 2010). Given this phenomenon, the question arises: Why do teams employ structure?

**FIGURE 1:**
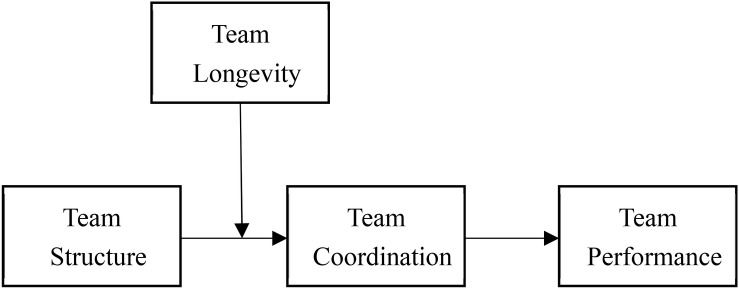
Theoretical model.

The literature argues team structure is able to help teams by improving learning (e.g., Bunderson and Boumgarden, 2010; Bresman and Zellmer-Bruhn, 2013) and coordination (e.g., transactive memory systems, Austin, 2003; Ren and Argote, 2011). However, other studies suggest that team structure may hurt performance by reducing creativity or team learning (e.g., Edmondson, 2003; Hirst et al., 2011). It is noted that a more basic task for teams is to integrate individual members’ work into the team’s goals (Olson, 1965; Gruenfeld and Tiedens, 2010), so we propose that team structure is more likely to play a coordinated role in teams rather than a promotor or an interrupter of creativity or learning. This study directly tested and proposed that team structure helps with teamwork coordination mechanism, which improves team performance. We also proposed the positive effects of team structure on team coordination and performance are stronger in the stage with a higher need of coordination (i.e., a high level of team longevity). In addition, we also compared the effect of team coordination and team learning the relationship between team structure and team performance. Our theoretical model is described in Figure 1.

This study advances relevant research in two ways. First, we contribute to team structure research by identifying the effect of team structure on team coordination. Most studies on team structure unpack the association between team structure and team learning (e.g., Bunderson and Boumgarden, 2010; Bresman and Zellmer-Bruhn, 2013). Our work extends the research by examining the effect of team structure on team coordination, and finds that structure can also improve coordination at team level. Secondly, this study highlights the importance of temporal factor on the effect of team structure. The results of this study show that the effect of team structure varies across the teams with different level of team longevity, such that team structure promotes team coordination when team longevity is high rather than low. This finding extends conditional context research on team structure.

## Theory and Hypothesis

Team structure refers to the extent to which the division of labor (specialization), leadership roles within the team (hierarchy), work routines, priorities and procedures (formalization) are clearly defined and understood by the team members (Bunderson and Boumgarden, 2010). Team structure is defined as a single-dimension construct comprised of these three elements. More specifically, a highly structured team has a clear division of labor, hierarchical role differentiation, and rules or procedures to guide the team’s work (Bunderson and Boumgarden, 2010).

In addition, team structure can be designed and shaped by outside superiors (e.g., organizational leaders, managers) (Stewart, 2006) and also by team members (Bresman and Zellmer-Bruhn, 2013). Normally, organizations provide structural frameworks for teams or team subunits, and the teams can develop and adjust their structures based on these frameworks (Birkinshaw, 2008). Thus, team structure is more informal than organizational structure and operates at a team level. Following the concept of organization structure, the concept of team structure describe the degree to which task related activities are structured within team (Bunderson and Boumgarden, 2010). Therefore team structure differs from the concepts that emphasize cognitive or knowledge structure within team (e.g., shared mental model, transactive memory system).

As previously noted, the literature on team structure generally investigates its effect on learning (e.g., Bunderson and Boumgarden, 2010; Bresman and Zellmer-Bruhn, 2013) and creativity (e.g., Edmondson, 2003; Hirst et al., 2011). However, the findings are inconsistent and paradoxical. Some studies suggest that team structure provides psychological safety, providing a safe and predicable environment for team members, which in turn benefits team learning (e.g., Bresman and Zellmer-Bruhn, 2013). However, other studies suggest that team structure may constrain team members’ creativity because of low participation and lack of team member autonomy (Hirst et al., 2011). This dispute derives in part from the heated discussion among organizational structure scholars on whether organizational structure benefits or harms innovation (e.g., Thompson, 1965; Pierce and Delbecq, 1977; Yang et al., 2015; Keum and See, 2017).

However, there is scant research explores the effect of team structure on coordination. Studies have investigated the association between organizational structure and organizational coordination (e.g., Thompson, 1965; Carzo and Yanouzas, 1969; Martinez and Jarillo, 1989; Adler and Borys, 1996). Studies on transactive memory system (TMS) suggests that coordination is an element of team cognitive or knowledge structure (e.g., Lewis, 2003; Lewis et al., 2005), however this definition of structure differs with the traditional definition of structure that emphasizes task and order structure (e.g., Thompson, 1965; Pierce and Delbecq, 1977). Therefore, this study explores the effect of team structure on team coordination and team performance.

### Team Structure, Team Coordination, and Team Performance

A basic problem with teamwork is how to integrate individual work into collective goals (Espinosa et al., 2007; Gruenfeld and Tiedens, 2010). Coordination refers to the process that temporally integrates individual team member work into collective goals (e.g., Faraj and Xiao, 2006). Okhuysen and Bechky (2009) review relevant studies on coordination and contend that coordination functions by creating three conditions: accountability, predictability, and common understanding. We suggest that team structure supports all three conditions, so team structure can benefit team coordination.

First, team structure clearly defines each team member’s role and tasks using specialization, hierarchy and formalization (Bunderson and Boumgarden, 2010). Just how these roles and tasks are fulfilled can be tracked and adjusted by members of the hierarchy (Tarakci et al., 2016). A body of studies suggests that hierarchy in a team tends to decrease uncertainty in interpersonal interactions by establishing order and rank differentiation (e.g., Magee and Galinsky, 2008; Halevy et al., 2011). Moreover, team hierarchy has been found to benefit intrateam coordination (e.g., Halevy et al., 2012). Second, when roles, tasks, task sequences, and schedules have been clearly specified by a team’s structure, team members are likely to know others’ tasks and plans. Therefore team members can predict what their teammates are doing and which activities they will respond to in certain situations and in what sequence (Bunderson and Boumgarden, 2010; Bresman and Zellmer-Bruhn, 2013). Therefore, teamwork becomes a more predictable process under a high level of team structure. Third, studies suggest that routines and rules can provide team members with information cues about what individual tasks should be done in certain situations so as to accomplish the team’s collective tasks (e.g., Cohen et al., 2014; Pentland and Hærem, 2015). Studies also indicate that formalization helps team members to establish a shared understanding about how to organize individual work to achieve collective goals (e.g., Feldman and Rafaeli, 2002). Moreover, hierarchy may also facilitate shared understanding; this occurs because hierarchy helps to establish shared behavioral expectations for members of different ranks in the hierarchy (Halevy et al., 2011). Empirical studies have shown that hierarchy is positively related to team member schema agreement (e.g., Rentsch and Klimoski, 2001). In sum, we propose the following hypothesis:

Hypothesis 1: Team structure will positively relate to team coordination.

Team coordination is defined as the activities that temporally manage discrete tasks and coordinate these tasks into team work flow (e.g., Marks et al., 2001; Kozlowski and Bell, 2013; Li and Liao, 2014). Studies find that team coordination is an important team process as it enables teams to function effectively (e.g., Marks et al., 2001; Kozlowski and Bell, 2013). Teams are able to integrate various individual teammate tasks with team goals through coordination of efforts so that team members can contribute to collective goals rather than individual interests and purposes (Kozlowski and Bell, 2013). Under a high level of coordination, the team’s information, resources and individual members’ skills and abilities can more readily be integrated into an efficient temporal workflow pace and task sequence, and ultimately enhance performance (Li and Liao, 2014). Indeed, empirical studies have shown that team coordination has a positive effect on team performance (e.g., Li and Liao, 2014; Reagans et al., 2016; Sui et al., 2016). Based on this, we propose our second hypothesis.

Hypothesis 2: Team coordination will mediate the relationship between team structure and team performance.

### Team Longevity: A Moderating Effect

Some studies suggest that a team’s focus often changes over time, and that teams are likely to explore and experiment with teamwork approaches and procedures when teams are initially set up, whereas teams tend to complete their team tasks more effectively when they are familiar with their teammates (i.e., due to longer time working together) and each other’s tasks (Gersick, 1988; Chang et al., 2003; Koopmann et al., 2016). Small group development model also support that the requirement for structure varies across the teams with different stages of development (Tuckman, 1965; Tuckman and Jensen, 1977). That is, teams only need a ground or loose structure to keep freedom to explore or test how to complete tasks and how to work with peers in early stages of development (i.e., forming and storming), but they embrace structure to effectively complete group task and coordinate team members in late stage of development (i.e., norming and performing) (Tuckman, 1965; Worchel, 1996; Bonebright, 2010). Team longevity refers to the length of time and shared experience that team members have been working together (Katz, 1982). Therefore, the relationship between team structure and team coordination is likely be moderated by team longevity.

The positive effect of team structure on team coordination may be stronger for teams with greater longevity vs. a low longevity level (young teams). As noted above, team structure provides clear and defined roles, routines and ranks for team members, and thus helps to improve team coordination. Under a low level of team longevity, teams tend to understand new situations (e.g., new tasks, changes in schedule, new goals), and test the way of groupwork and interpersonal relationship (Worchel, 1996; Bonebright, 2010). In other words, when a team is in the initial stage, team members focus on exploring and finding the best method to perform their tasks and work together as a team (Tuckman and Jensen, 1977; Chang et al., 2003). This initial activity is filled with uncertainty and complexity, therefore routines, responsibilities and rank differentiation are not able to effectively organize this exploration process (Gersick and Hackman, 1990; Sieweke and Zhao, 2015). In this stage, team members even try to resist team structure, because team structure constrains their exploration and forces them behave in a new way (Tuckman and Jensen, 1977; Bonebright, 2010). In support of this view, many studies have suggested that a tight structure may stifle members’ creativity (e.g., Hirst et al., 2011; Yuan and Zhou, 2015). Thus, the positive effect of team structure on team coordination may be limited in young (low-longevity) teams. The research on development of small group also found that team members tend to resist structure in initial stage but prefer structure in late development stage (Maples, 1988).

Conversely, teams with high longevity (e.g., mature teams) face less complexity and uncertainty because team members are familiar with their own and others’ tasks and their teammates in general (Gersick, 1988; McGrath, 1991). When team longevity is high, teams focus on how to compete tasks most effectively, such that team members are task orientated and seek high productivity (e.g., Tuckman and Jensen, 1977; Gersick, 1988; Maples, 1988). Under this condition, the effect of team structure is likely to be greater. A high level of team structure is likely to help teams define team members’ roles and ranks, divide the labor, and establish routines and plans for effective, collective work (Bunderson and Boumgarden, 2010). In such cases, team members have a clear understanding of their responsibilities, team goals and the team’s work schedule. Therefore, teams can effectively integrate individual work and improve efficiency in the implementation process. Based on these findings, we pose our third hypothesis.

Hypothesis 3: Team longevity will moderate the relationship between team structure and team coordination such that this relationship will be stronger when team longevity is high.

## Overview of Our Field Studies

We carried out two field studies to test our three hypotheses. We first investigated 57 engineering teams to test all three hypotheses. Next, we replicated the results of field study 1 in field study 2 using a larger sample of 67 work teams.

## Study 1

### Sample

The study 1 investigated 72 engineering teams in 20 manufacturing company located in Hangzhou, China. We sent questionnaires to participants and collected questionnaires face to face. Of these, 65 teams with 63 supervisors returned questionnaires to us (response rate = 87.5%) and 286 team members returned questionnaires to us (response rate = 79.01%). To address common method bias concerns (Podsakoff et al., 2003), the dependent variable (i.e., team performance) was assessed by team supervisors and other variables – team structure, team coordination and team longevity – were assessed by the team members. The teams included in the data analysis if more than half of the team members completed questionnaires. Nine teams were excluded because less than half of the team members completed the questionnaires. Ultimately, 56 teams with 56 team supervisors and 242 team members were included in further analysis. The average age of team members was 30.95 (*SD* = 6.07), 68% were male, and 73% had a bachelor’s degree or above.

### Measures

Established scales were employed to measure our variables. Because the scales were originally developed in English, the transition/back-transition procedure (Brislin, 1980) was employed to translate scales from English to Chinese. The specific measures are described next.

#### Team Structure

A 5-item Likert scale adopted from Bunderson and Boumgarden (2010) was employed to measure team structure. This scale contains three elements of team structure – specification, hierarchy and formalization. One example item for specification is “Each team member has their particular area of specialty in the team”; an example of a hierarchy question is “There is a clear leader who directs what we do in the team”; and an example item for formalization is “We follow a very structured work schedule in the team.” Team members were required to rate these five items on 7-point Likert scales (1 = totally disagree to 7 = totally agree). Cronbach’s α for this scale was 0.89.

#### Team Coordination

A 5-item Likert scale adopted from Lewis (2003) was used to measure team coordination. Two example questionnaire items: “The team worked together in a well-coordinated fashion” and “The team had very few misunderstandings about what to do.” Team members were asked to evaluate these five items on 5-point Likert scales (1 = totally disagree to 5 = totally agree). Cronbach’s α for this scale was 0.81.

#### Team Performance

Team performance was measured by 3-item Likert scale adapted from Ancona and Caldwell (1992). Examples of these items: “The work efficiency in our team is satisfying” and “The work quality in our team is satisfying.” Team supervisors were required to rate these three items on 7-point Likert scales (1 = totally disagree to 7 = totally agree). Cronbach’s α for this scale was 0.86.

#### Team Longevity

Team longevity is calculated by averaging team members’ team tenure (Katz, 1982). Team members were required to report the date that they started working on their current teams. Then team tenure was calculated for each team member. Finally, we averaged team tenure as team longevity for each team.

#### Control Variables

Several variables likely to affect team performance were controlled in study 1. First, team size has been found to affect the relationship between team processes and team performance (e.g., Lepine et al., 2008), so we controlled for team size. Second, given that many studies suggest that information-based diversity has a critical influence on team processes and performance (e.g., Pelled et al., 1999; van Knippenberg et al., 2004), we controlled for education level diversity and education background (i.e., study majors). Third, as a body of research shows, leaders play important roles in team functions (e.g., Giessner et al., 2013; Tost et al., 2013), and team leaders’ competences and experiences can affect team performance (e.g., Sieweke and Zhao, 2015), so team leaders’ education level and organizational tenure were controlled.

#### Data Aggregation

The justification of data aggregation needed verification. First, we examined interrater reliability using r_wg_, as recommended by James et al. (1984). The mean r_wg_ was 0.88 for team structure, and 0.90 for team coordination. Both values were above 0.7, which is a common acceptable cutoff value (George, 1990). We also calculated the intraclass correlation coefficients as suggested by Bliese (2000). The results show that ICC(1) was 0.12, and ICC(2) was 0.36 for team structure. Regarding team coordination, ICC(1) was 0.13, and ICC(2) was 0.40. The values of ICC(1) for both team structure and team coordination were over 0.12, which is considered the median value of ICC(1) for most team research (James, 1982). However, even though our results of ICC(2) were similar to team research with small samples (e.g., Koopmann et al., 2016; Mitchell et al., 2012), these values of ICC(2) were rather low in study 1.

### Results

Table 1 shows the results of descriptive statistics for all variables. In hypothesis 1, we proposed that team structure will positively relate to team coordination, and in hypothesis 2, we proposed that team coordination will mediate this relationship. We further tested our hypotheses using hierarchical regression. Note that all predictors in the regressions were mean-centered to eliminate the likelihood of multicollinearity.

**TABLE 1:** Means, standard deviation and correlates between variables (study 1).

Variable	Mean	*SD*	1	2	3	4	5	6	7	8	9
1 Team size	6.88	2.62	−								
2 Education background Diversity	0.33	0.28	0.24	−							
3 Education level diversity	0.31	0.25	0.17	0.37**	−						
4 Leader education level	3.07	0.76	0.15	−0.31*	–0.19	−					
5 Leader organizational tenure	8.79	6.17	–0.08	0.15	0.07	−0.28*	−				
6 Team structure	5.55	0.64	–0.15	0.20	0.05	0.01	–0.03	−			
7 Team longevity	3.36	2.74	–0.01	0.03	–0.01	−0.32*	0.50**	0.08	−		
8 Team coordination	3.78	0.40	–0.14	–0.06	–0.23	0.21	–0.06	0.62**	0.07	−	
9 Team performance	4.97	0.82	–0.01	–0.05	–0.11	–0.06	–0.06	0.28*	0.09	0.39**	−

*n = 56.* *p < 0.05; **p < 0.01.

As shown in Table 2, the positive regression coefficient of team structure on team coordination was significant after we controlled all control variables (*b* = 0.40, *p* < 0.01). Hence, hypothesis 1 was supported. We employed Baron and Kenny’s method (Baron and Kenny, 1986) to test the mediation effect of team coordination between a team’s structure and its performance. First, as model 2 represents, we found a positive and significant relationship between team structure and team performance in the regression analysis (*b* = 0.43, *p* < 0.05). Second, as explained above, the relationship between team structure and coordination was also positive and significant. Third, when team coordination was entered in model 3, team coordination was positively associated with team performance (*b* = 0.77, *p* < 0.05), but the coefficient of team structure on team performance became non-significant (*b* = 0.12, *ns.*). As a result, hypothesis 2 was supported.

**TABLE 2:** The hierarchical regression results for team structure, team coordination, and team performance (study 1).

Variables	Team performance			Team coordination			
		
	M1	M2	M3	M4	M5	M6	M7
Team size	0.01	0.03	0.04	–0.03	–0.00	–0.01	–0.01
Education background diversity	–0.10	–0.39	–0.33	0.19	–0.08	–0.06	–0.08
Education level diversity	–0.38	–0.39	–0.13	–0.34	–0.34	–0.33	–0.30
Team leader education level	–0.12	–0.17	–0.23	0.12	0.08	0.09	0.14
Team leader organizational tenure	–0.10	–0.01	–0.01	–0.00	0.00	–0.00	–0.00
Team structure		0.43*	0.12		0.40**	0.39**	0.42**
Team longevity						0.02	0.02
Team structure × Team longevity							0.08*
Team coordination			0.77*				
*F*	0.43	1.46	1.90^+^	1.20	7.60**	6.43**	6.64**
*R*^2^	0.02	0.12	0.20	0.12	0.48	0.48	0.53
Δ*R^2^*		0.10*	0.08*		0.36**	0.01	0.05*

*n = 56. ^+^p < 0.10;* *p < 0.05; **p < 0.01.

**FIGURE 2:**
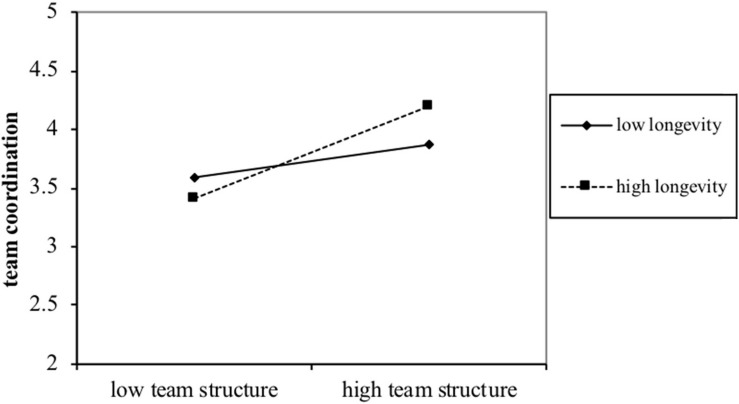
The moderation effect of team longeivty on the relationship between team strcture and team performance (study 1).

We assumed that team longevity moderates the relationship between team structure and team coordination in hypothesis 3. As shown in Table 2, the interactive term between team structure and longevity is significant (*b* = 0.08, *p* < 0.05) as shown in model 5. We further tested this moderated effect with sample slope tests (Dawson, 2014). Figure 2 represents the relationship between team structure and performance across different levels of team longevity. Though the relationship between team structure and team performance was positive and significant for both high longevity (1 SD above mean) and low longevity (1 SD below mean), this relationship was stronger when team longevity was high (*b* = 0.63, *p* < 0.01) vs. low (*b* = 0.22, *p* < 0.05).

Finally, we tested the moderated mediation effects using the bootstrapping method suggested by Edwards and Lambert (2007). The results, summarized in Table 3 show that although the indirect effect of team structure on team performance via team coordination is positive and significant (at a 95% confidence interval that does not include 0) at all levels of team longevity, the effect size is greater for a high longevity level (1 SD above mean) than a low level of longevity (1 SD below mean). We further tested this difference of effect size using bootstrapping; the results suggested that the indirect effect of team structure on team performance is stronger when team longevity is high vs. low (at a 95% confidence interval that does not include 0). Taken together, hypothesis 3 was supported in study 1.

**TABLE 3:** The bootstrap results for moderated mediation effects – team coordination as mediator and team longevity as moderator (study 1).

Team longevity	Indirect effect	BCaL95	BCaU95
Low	0.17	0.00	0.50
Average	0.33	0.02	0.66
High	0.49	0.04	0.98
Diff (high vs. low)		0.02	0.81

**Bootstrapping sample = 5,000.**

## Study 2

Studies have found that team structure enhances team performance through the process of team learning (e.g., Bunderson and Boumgarden, 2010; Bresman and Zellmer-Bruhn, 2013). In a similar vein, we suggest that team structure affects team performance mainly through the team coordination process. Team learning enables team members to reflect, experiment and explore (Edmondson, 1999), whereas team coordination stimulates team members to integrate dispersed work (Kozlowski and Bell, 2013). Therefore, it is useful to compare the mediating effect of team coordination between team structure and team performance on team learning. In study 1, we only tested the coordination mechanism between team structure and team performance. To compare the mediated effect of team coordination and team learning on the relationship between team structure and team performance, we investigated both the coordination and learning mechanism in study 2.

### Sample

We investigated 450 employees with 80 team supervisors. Of these, 413 employees returned questionnaires to us (response rate = 91.8%) as did 75 supervisors (response rate = 93.8%). As in study 1, teams of which team supervisors and more than 50% of employees returned valid questionnaires were included in the data analysis; so six teams were excluded in study 2. In addition, two team were also excluded because of its small team size (i.e., team size = 2). Finally, 67 teams with 348 team members were used in further analysis in study 2. These teams included 8 RandD teams (12%), 11 financial management teams (16%), 9 marketing teams (13%), 25 human resource management teams (37%), 9 project teams (13%), and 5 others (8%). Of the 348 team members, the average age was 29.71 (*SD* = 5.63); 42% were male, 82% had a bachelor’s degree or above.

### Measures

We employed similar procedures and measurements that we used in study 1 to measure variables in study 2. That is, in study 2, we adopted the same scales and calculations as in study 1 to measure team structure, team coordination, team performance and team longevity. First, team members were required to assess team structure in a 5-item Likert scale adopted from Bunderson and Boumgarden (2010) (Cronbach’s α = 0.86), and to assess team coordination in a 5-item Likert scale adopted from Lewis (2003) (Cronbach’s α = 0.89). Second, team supervisors rated team performance in a three-item scale adapted from Ancona and Caldwell (1992) (Cronbach’s α = 0.84). Team longevity was calculated by averaging team tenure (Katz, 1982). Additionally, team learning was measured using a 7-item Likert scale adopted from Edmondson (1999) (Cronbach’s α = 0.84). Example items: “Our team regularly takes time to figure out ways to improve our team’s work processes” and “Team members in this team often speak out to test assumptions about issues under discussion.”

#### Data Aggregation

To check the justification of data aggregation, interrater reliability (James et al., 1984), and intraclass correlation coefficients (Bliese, 2000) were calculated in study 2. The results showed that mean r_wg_ is 0.88 for team structure, 0.88 for team learning, and 0.84 for team coordination. With respect to intraclass correlation coefficients, ICC(1) was 0.16, and ICC(2) was 0.47 for team structure; ICC(1) was 0.20, and ICC(2) was 0.54 for team learning; and ICC(1) was 0.12, and ICC(2) was 0.39 for team coordination. Though the values of ICC were relatively low for team coordination, the high value of r_wg_ indicates strong within-group agreement.

#### Control Variables

Consistent with study 1, in study 2, we controlled for team size, team education (subject area) diversity, education level diversity, team leader’s education level and organizational tenure.

### Results

Table 4 shows the results of descriptive statistics and correlations between both variables. Next, we employed a hierarchical regression analysis to test our hypotheses. To deal with multicollinearity concerns, all predictors were mean-centered before entered into the regressions.

**TABLE 4:** Means, standard deviation and correlates between variables (study 2).

Variable	Mean	*SD*	1	2	3	4	5	6	7	8	9
1. Team size	6.60	3.84	−								
2. Education background diversity	0.51	0.29	0.03	−							
3. Education level diversity	0.34	0.25	0.08	0.11	−						
4. Leader education level	3.28	0.63	0.08	–0.24	–0.11	−					
5. Leader organizational tenure	4.08	3.07	0.19	0.00	0.02	0.15	−				
6. Team structure	4.25	0.40	0.20	0.19	–0.17	0.24	0.20	−			
7. Team longevity	2.03	1.56	0.19	0.15	–0.13	0.20	0.45**	0.13	−		
8. Team coordination	3.99	0.38	0.02	0.18	0.05	0.20	0.23	0.73**	0.17	−	
9. Team learning	3.87	0.40	0.05	0.18	0.01	0.16	–0.00	0.50**	0.07	0.48**	−
10. Team performance	3.89	0.48	–0.10	0.05	–0.11	–0.19	0.02	0.18	–0.04	0.35**	0.16

*n = 67 for most of variables, n = 65 for variable 10 because of missing data.* *p < 0.05; **p < 0.01.

We proposed that team structure will positively correlate to team coordination in hypothesis 1. Table 5 shows the results of our hierarchical regression. After controlling for team size, education background diversity, education level diversity, team leader’s education level and organizational tenure, we found a positive and significant coefficient for team structure when it was added in model 6 (M6) (*b* = 0.77, *p* < 0.01). Therefore hypothesis 1 was supported.

**TABLE 5:** Hierarchical regression results between team structure, team coordination and team performance (study 2).

Variables	Team performance^a^				Team coordination^b^			
		
	M1	M2	M3	M4	M5	M6	M7	M8
Team size	–0.01	–0.02	–0.01	–0.01	–0.00	–0.02	−0.02*	−0.02*
Education background diversity	0.02	–0.15	–0.12	–0.12	0.34	–0.03	–0.05	–0.04
Education level diversity	–0.24	–0.15	–0.32	–0.33	0.09	0.28*	0.30*	0.29*
Leader education level	–0.16	−0.23*	−0.24*	−0.24*	0.15	0.02	0.00	0.02
Leader organizational tenure	0.01	0.01	–0.00	–0.00	0.02	0.01	0.01	0.01
Team structure		0.36*	–0.17	–0.19		0.77**	0.78**	0.84**
Team longevity							0.03	0.01
Team structure × Team longevity								0.15*
Team coordination			0.69**	0.68**				
Team learning				0.04				
*F*	0.74	1.37	2.55*	2.20*	1.87	15.23**	13.33**	12.90**
*R*^2^	0.06	0.13	0.25	0.25	0.14	0.62	0.63	0.65
Δ*R*^2^	0.74	0.07*	0.12**	0.001	0.14	0.48**	0.01	0.03*

*^*a*^n = 65. ^*b*^n = 67.* *p < 0.05; **p < 0.01.

Next, we used the procedures recommended by Baron and Kenny (1986) to examine hypothesis 2, which proposed that team coordination will mediate the relationship between team structure and performance. As shown in models 2 and 5, team structure had a positive and significant relationship with team performance (*b* = 0.36, *p* < 0.05) and team coordination. However, the relationship between team structure and team performance became insignificant (*b* = -0.17, *ns*) when team coordination was added in model 3 (M3). And the relationship between team coordination and team performance remained positive and significant (*b* = 0.69, *p* < 0.01) after controlling for team structure and control variables. As such, hypothesis 2 was supported.

In addition, we also compared the indirect effect of team learning and team coordination. When we entered team coordination and team learning into model 4 (M4), the effect of team coordination remained significant (*b* = 0.68, *p* < 0.01), whereas the effect of team learning was nonsignificant (*b* = 0.04, *n.s.*). The result of bootstrapping (bootstrapping sample = 20,000) also showed that the indirect effect of team coordination (effect = 0.48, 95% CI [0.10, 0.95]) was stronger than team learning (effect = 0.02, 95% CI [-0.26, 0.26]). Even the indirect effect of team learning was also nonsignificant (effect = 0.07, 95% CI [-0.14, 0.33]) when it solely played the role of mediator. Thus, the indirect effect of team coordination on the relationship between team structure and team performance is stronger than team learning.

**FIGURE 3:**
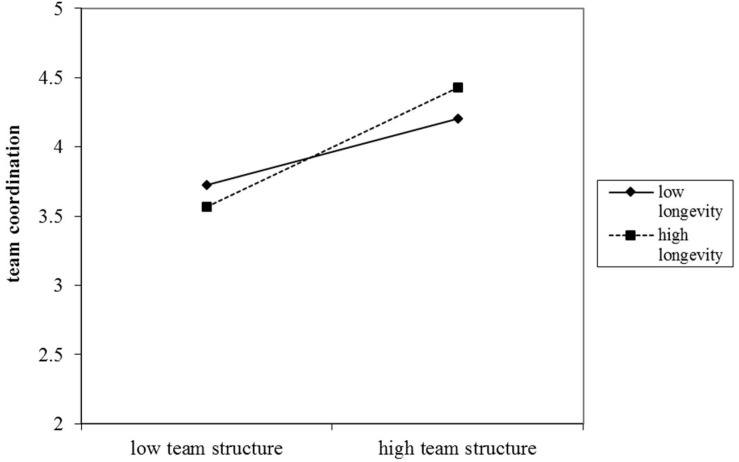
Study 2: The moderation effect of team longeivty on the relationship between team strcture and team performance (study 2).

Hypothesis 3 proposed that team longevity will moderate the relationship between team structure and team coordination. As expected, the interactive term between team structure and team longevity was positive and significant (*b* = 0.15, *p* < 0.05) when it was added in model 7 (M7). To further test this moderation effect, we conducted and then graphed simple slope tests as suggested by Dawson (2014). The results indicated that the relationship between team structure and team coordination is positive and significant at both high levels of longevity (1 *SD* above mean, *b* = 1.08, *p* < 0.01) and low levels of longevity (1 *SD* below mean, *b* = 0.60, *p* < 0.01). However, as shown in Figure 3, this positive relationship was steeper when team longevity was high. Overall, hypothesis 3 was supported.

We tested this moderated mediation effect using the bootstrapping method recommended by Edwards and Lambert (2007). As Table 6, the results of bootstrapping reports, the indirect effect of team structure on team performance via team coordination was positive and significant (95% confidence interval does not include 0) at all three levels of team longevity. Nevertheless, the effect size of the indirect effect was greater for teams with high levels of team longevity (1 SD above mean) than for low-longevity teams (1 SD below mean). This difference of effect size was significant (at 95% confidence interval that does not include 0). These results supported Hypothesis 3. Finally, we compared the effect of team coordination and team learning using the bootstrapping method with 5000 bootstrap samples. The results show that the indirect effect via team coordination was significant at 95% confidence intervals (effect = 0.53, [0.06, 0.95]), whereas the indirect effect via team learning was nonsignificant (effect = 0.02, [-0.25, 0.25]).

**TABLE 6:** The bootstrap results for moderated mediation effects – team coordination as mediator and team longevity as moderator (study 2).

Team longevity	Indirect effect	BCaL95	BCaU95
Low	0.40	0.10	0.82
Average	0.59	0.11	1.08
High	0.77	0.15	1.56
Diff (high vs. low)		0.01	1.01

**Bootstrapping sample = 5,000.**

## Discussion

Though many studies suggest that team structure mainly influences team performance through team learning or creativity (e.g., Bunderson and Boumgarden, 2010; Bresman and Zellmer-Bruhn, 2013), we proposed that team structure can act as a coordinating mechanism, which in turn improves team task coordination and ultimately, boosts team performance. We conducted two field studies to test our three hypotheses. The results show that team structure benefits team performance via team coordination. Moreover, we proposed that the effect of team structure on performance via coordination is likely to be based on team longevity levels. This hypothesis was also supported in our two field studies, such that the positive relationship between team structure and team coordination is stronger when team longevity is high.

This study contributes to relevant literature in two ways. First, we contribute to the team structure literature by finding that team structure is likely to improve team performance by supporting team coordination. Most studies on team structure focus mainly on its effect on team learning (e.g., Bunderson and Boumgarden, 2010; Bresman and Zellmer-Bruhn, 2013) and creativity (e.g., Hirst et al., 2011). We explored whether team structure can also help coordinate team members and team tasks, a critical issue in team work literature. Our results indicate that team structure can also benefit team performance by improving team coordination. Moreover, we compared the mediated effect of team coordination with team learning on the relationship between team structure and team performance in study 2, and found the team coordination effects was stronger than the team learning effect. Kozlowski and Bell (2013) suggest that team learning is a typical cognitive mechanism, and team coordination is a vital behavioral mechanism that influences team effectiveness. Most research on team structure investigates its effect through cognitive mechanisms (e.g., learning and creativity) (e.g., Bunderson and Boumgarden, 2010; Hirst et al., 2011). However, our findings show that team structure mainly influences team performance through behavioral (i.e., coordination) rather than cognitive mechanisms.

Secondly, this study advances research on team structure by highlighting the importance of temporal factors on team functioning. Studies suggest that the effect of team structure depends on organizational structure (e.g., Bresman and Zellmer-Bruhn, 2013) and individual goal orientation (e.g., Hirst et al., 2011). In other words, drawing from these previous studies (e.g., Hirst et al., 2011; Bresman and Zellmer-Bruhn, 2013), the effect of team structure may be influenced by the organizational context and individual personalities on the team. However, they overlook the effect of team context. Many researchers claim that time (i.e., the time that the team has been working together is a basic factor that shapes team processes and functions (e.g., Gersick, 1988; Chang et al., 2003; Harrison et al., 2003), however, team structure research fails to adequately explore the temporal factor. We found that team longevity can moderate the effect of team structure on team coordination. In other words, the coordinated effect of structure may vary across a team’s longevity. We found that the effect of team structure is also contingent on team context, especially influenced by the team’s longevity or development stage.

## Practical Implications

This study offers insights for managers. In responding to the challenges of a dynamic environment, many contemporary organizations employ a team-based flat structure rather than a department-based tall structure. Note that today’s teams are also more or less structured, so how best to cope with team structure is critical for managers. Drawing from the logics of organizational structure change, some managers may contend that team structure should be eliminated or attenuated to free team members. However, our findings suggest that these attempts to eliminate or attenuate team structure *may reduce the benefits of coordination and performance from team structure*. Team structure can effectively integrate individual work through establishing clear rules, procedure, and roles for team task, then team productivity and efficiency can be elevated. Team members’ goal may struggle with each other and be inconsistent with collective goal, and then lead to chaos and inefficiency within team. The problem of loss in team structure is likely to more salient in current organizations, in which a body of them have employed a flat structure at organizational level. It because the teams in these flat-structure organizations do not have substitute for team structure from organizational structure (Bresman and Zellmer-Bruhn, 2013). Moreover, we suggest that the positive effect of team structure on coordination is likely to be stronger for teams that have existed for a long time vs. newly formed or young team. Therefore, managers could make more structured arrangements to help teams establish routines and carry out action plans when task effectiveness or efficiency is necessary.

## Limitations

Like most empirical studies, this study has several limitations. First, as a cross-sectional study, we cannot make any causal inferences. Though no evidence supports that improved performance can lead to a high level of team structure, experimental studies are required to test our causal logics. Second, although we employed several methods to attenuate common method bias (e.g., assessing variables from different sources), we collected data in just at one time point. This process could create the problem of common method bias about time (Podsakoff et al., 2012). Future studies could collect data at different points of time.

## Conclusion

Although most relevant research on team structure focuses on its effect on learning or creativity (e.g., Bunderson and Boumgarden, 2010; Hirst et al., 2011), we propose that team structure is likely to help teams address a basic problem of team function, that is, coordination. To test our hypotheses, we conducted two field studies with 56 and 67 work teams. We found that higher levels of team structure, achieved by improving team coordination, improved team performance. In addition, we found that the relationship between team structure and team coordination is moderated by team longevity, such that the positive relationship between team structure and coordination is stronger when the team has worked together for a longer time period, that is, when team longevity is high.

## Data Availability Statement

The raw data supporting the conclusions of this article will be made available by the authors, without undue reservation, to any qualified researcher.

## Ethics Statement

The study of “How Team Structure Can Enhance Performance: Team Longevity’s Moderating Effect and Team Coordination’s Mediating Effect” involving human participants was reviewed and approved by the Ethics Committee of Department of Psychology and Behavioral Sciences in Zhejiang University (No. 201902), China. Written informed consent to participate in this study was provided by the participants.

## Author Contributions

HJ completed the research idea, data analysis, and writing of the draft. JY developed the theoretical framework and the theoretical hypotheses together with HJ, and discussed the revisions of the manuscript. Both authors contributed to the article and approved the submitted version.

## Conflict of Interest

The authors declare that the research was conducted in the absence of any commercial or financial relationships that could be construed as a potential conflict of interest.
